# Suppressed *Helicobacter pylori*-associated gastric tumorigenesis in *Fat-1* transgenic mice producing endogenous ω-3 polyunsaturated fatty acids

**DOI:** 10.18632/oncotarget.11261

**Published:** 2016-08-12

**Authors:** Young-Min Han, Kyung-Jo Kim, Migyeung Jeong, Jong-Min Park, Eun-Jin Go, Jing X Kang, Sung Pyo Hong, Ki Baik Hahm

**Affiliations:** ^1^ CHA Cancer Prevention Research Center, CHA Cancer Institute, CHA University, Seoul, Korea; ^2^ Department of Gastroenterology, University of Ulsan, Seoul Asan Medical Center, Seoul, Korea; ^3^ Laboratory for Lipid Medicine and Technology, Massachusetts General Hospital, Harvard Medical School, Boston, USA; ^4^ Department of Gastroenterology, CHA Bundang Medical Center, Seongnam, Korea

**Keywords:** Fat-1 transgenic mice, COX-2, 15-PGDH, ω-3 PUFAs, anti-proliferation

## Abstract

Dietary approaches to preventing *Helicobacter pylori* (*H. pylori*)-associated gastric carcinogenesis are widely accepted because surrounding break-up mechanisms are mandatory for cancer prevention, however, eradication alone has been proven to be insufficient. Among these dietary interventions, omega-3-polyunsaturated-fatty acids (ω-3 PUFAs) are often the first candidate selected. However, there was no trial of fatty acids in preventing *H. pylori*-associated carcinogenesis and inconclusive results have been reported, likely based on inconsistent dietary administration. In this study, we developed an *H. pylori* initiated-, high salt diet promoted-gastric tumorigenesis model and conducted a comparison between wild-type (WT) and *Fat*-1-transgenic (TG)-mice. Gross and pathological lesions in mouse stomachs were evaluated at 16, 24, 32, and 45 weeks after *H. pylori* infection, and the underlying molecular changes to explain the cancer preventive effects were investigated. Significant changes in: i) ameliorated gastric inflammations at 16 weeks of *H. pylori* infection, ii) decreased angiogenic growth factors at 24 weeks, iii) attenuated atrophic gastritis and tumorigenesis at 32 weeks, and iv) decreased gastric cancer at 45 weeks were all noted in *Fat-*1-TG-mice compared to WT-mice. While an increase in the expression of Cyclooxygenase (COX)-2, and reduced expression of the tumor suppressive 15-PGDH were observed in WT-mice throughout the experimental periods, the expression of Hydroxyprostaglandin dehydrogenase (15-PGDH) was preserved in *Fat-1-*TG-mice. Using a comparative protein array, attenuated expressions of proteins implicated in proliferation and inflammation were observed in *Fat*-1-TG-mice compared to WT-mice. Conclusively, long-term administration of ω-3 PUFAs can suppress *H. pylori*-induced gastric tumorigenesis through a dampening of inflammation and reduced proliferation in accordance with afforded rejuvenation.

## INTRODUCTION

The International Agency for Research on Cancer defined *Helicobacter pylori* (*H. pylori*) as a class I carcinogen [1] as *H. pylori* caused gastric carcinogenesis [2] and the eradication of *H. pylori* prevented metachronous gastric cancer (MGC) after endoscopic resection of early gastric cancer [3]. During carcinogenesis, *H. pylori* provoked gastric inflammation, oxidative stress, and several harmful events including genetic and epigenetic pathways [4], after which its eradication can be a solution for prevention. However, in intervention trials studying gastric cancer prevention, *H. pylori* eradication did not prevent MGC in patients undergoing endoscopic submucosal dissection, have yielded rather disappointing results [5]. Therefore, non-microbial dietary intervention has been considered asan alternate eradication method or as a provision of surrounding break up to either remove mutagenic inflammation leading to cancer progression [6–9].

Generally, strategies to reduce the occurrence of gastric cancer, include improvement of sanitation, high intake of fresh fruits, safe food-preservation methods, and avoidance of smoking [10]. Additionally, mitigating the chronic inflammatory response associated with infectious disease has been recommended [11], including non-microbial dietary intervention or supplementation with phytoceuticals with the hope that these approaches may be an effective way of preventing cancer through long-term control of gastric inflammation [12]. Increased consumption of fatty fish or fish oil supplements containing anti-inflammatory ω-3 PUFAs is also an intriguing intervention since ω-3 PUFA has been shown to have a therapeutic role in inflammatory diseases such as rheumatoidarthritis, inflammatory bowel disease, asthma, cardiovascular, and neurodegenerative diseases [13–15] and various cancers by reducing the level of AA-derived eicosanoids and inflammatory cytokines, (including Interleukin (IL)-1, IL-2, IL-6, IL-8, Interferon (IFN)-γ,and Leukotriene B4) and Tumor necrosis factor-α, promoting anti-inflammatory activities [16, 17]. In spite of these achievements, the expected clinical impact of ω-3 PUFAs has been reduced, perhaps because applying ω-3 PUFA-containing diet scan bring considerable variations between the experimental groups [18, 19], resulting from an in consistent intake and uncertain purity of ω-3 PUFAs.

*Fat-*1 transgenic *(Fat-*1 TG) mice are capable of producing ω-3 fatty acids from ω-6 type fatty acids because of the transgenic over-expression of n-3 *desaturase* lead to abundant ω-3 PUFAs with reduced levels of ω-6 fatty acids in their organs and tissues without a dietary n-3 supply [20]. Here we used these mice to explore the effect of ω-3 PUFAs, especially the exact effect of ω-3 PUFAs on *H. pylori* associated gastric lesions, something that has never been studied before. We hypothesize that *Fat-*1 TG mice may be conferred a chemo-preventive benefit through anti-inflammatory or anti-mutagenic actions of ω-3 PUFAs. Using *H. pylori* infected mice, our study can provide important preclinical evidence that ω-3 PUFAs are efficient in attenuating *H. pylori*-associated gastritis and preventing gastric tumors and reveal molecular insights for the use of dietary ω-3 PUFAs in chemoprevention of *H. pylori*-associated gastric cancer.

## RESULTS

### Attenuated gastric inflammation in *Fat*-1 TG mice compared to WT mice at 16 weeks after *H. pylori* infection

**Figure 1:**
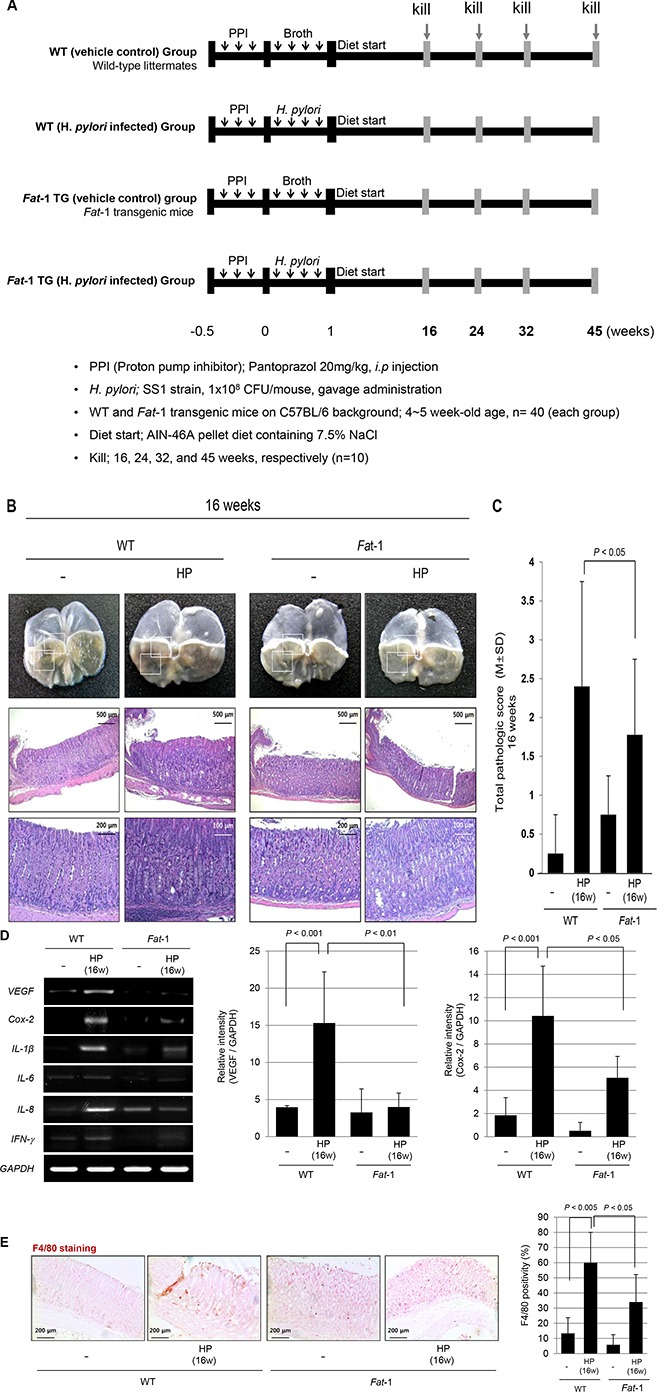
The influence of *Fat*-1 mice on *H. pylori*-initiated, high salt diet-promoted gastric tumorigenesis model at 16 weeks (**A**) Experimental scheme for *H. pylori*-associated gastritis model. The mice were grouped into four groups; non-infected normal in WT mice (*n* = 40), *H. pylori*-infected WT mice (*n* = 40), non-infected normal in *Fat*-1 TG mice (*n* = 40), and *H. pylori*-infected *Fat*-1 TG mice (*n* = 40), which were serially sacrificed at 16, 24, 32 and 45 weeks after *H. pylori*-initiated, high salt diet-promoted gastric tumorigenesis model. (**B**) Gross lesions and total pathological score according to group. *H. pylori* infection followed with high salt diet leads to some erosion, erythematous gastric mucosa at 16 weeks (box indicated the portion of pathologic analysis). At 16 weeks, *H. pylori* infections lead to significantly increased inflammatory cells infiltration at submucos and mucosa on body and antrum area and mucosal proliferations in WT mice, while these changes were significantly ameliorated in *H. pylori*-infected *Fat*-1 TG mice. (H&E stain, magnification, × 40, 100 and 200). (**C**) Total pathological scores among groups. (**D**) Expression of *VEGF*, *Cox-2*, *IL-1*β, *IFN-*γ, and *IL-6* mRNA is increased in *H. pylori*-infected WT mice group. On RT-PCR analysis for mucosal *Cox-2* mRNA expression, *Cox-2* was significantly increased after *H. pylori* infection (*P* < 0.001), but their expressions were significantly decreased in *Fat*-1 TG mice group (*P* < 0.05). The *VEGF* was also significantly increased after *H. pylori* infection (*P* < 0.001), but their expressions were significantly decreased in the *Fat*-1 mice group (*P* < 0.05). (**E**) The immunohistochemical changes of F4/80 denoting macrophage infiltrations according to groups (Magnification, × 100). *H. pylori* infection led to significant increased positivity in WT mice (*P* < 0.005), but significantly attenuated in *Fat*-1 TG mice (*P* < 0.05).

After16 weeks of *H. pylori* infection, there were no significant changes on gross lesions of resected stomachs of *H. pylori*-infected WT and *Fat*-1 TG mice (see Supplementary Figure S1). However, the gastric mucosa of WT mice were thicker than *Fat*-1 TG mice and this thickening was accompanied with intense infiltrations of inflammatory cells in sub-mucosal and mucosal layers. Compared to the non-infected group, marked inflammatory cells were observed in the sub-mucosa and mucosa in the stomachs of *H. pylori*-infected WT mice (Figure 1B), whereas these inflammatory cell infiltrations were significantly decreased in *Fat*-1 TG mice after *H. pylori* infection *(P* < 0.05, Figure 1C). Therefore, we compared the expression levels of inflammatory mediators between *H. pylori*-infected WT and *Fat*-1 TG mice. As seen in Figure 1D, the expression of cytokines known to be increasingly expressed at *H. pylori*-infected gastritis including Vascular endothelial growth factor (VEGF), Cyclooxygenase (Cox)*-2*, *IL-1*β, *IL-8*, *INF-γ*, and *IL-6*, [21], were all significantly increased in *H. pylori*-infected WT compared to non-infected vehicle control group (*P* < 0.001). However, their expression levels were all significantly decreased in *H. pylori*-infected *Fat-1* TG mice (*P* < 0.01). Since the sources of these inflammatory cytokines are macrophages and monocytes, we tested for macrophages using F4/80 immunohistochemical staining. As seen in Figure 1E, F4/80 expressions were significantly increased after *H. pylori* infection in WT mice, but significantly decreased in *Fat*-1 TG mice (*P* < 0.05).

### Mitigated chronic atrophic gastritis in *Fat*-1 TG mice compared to WT mice at 24 weeks after *H. pylori* infection

**Figure 2:**
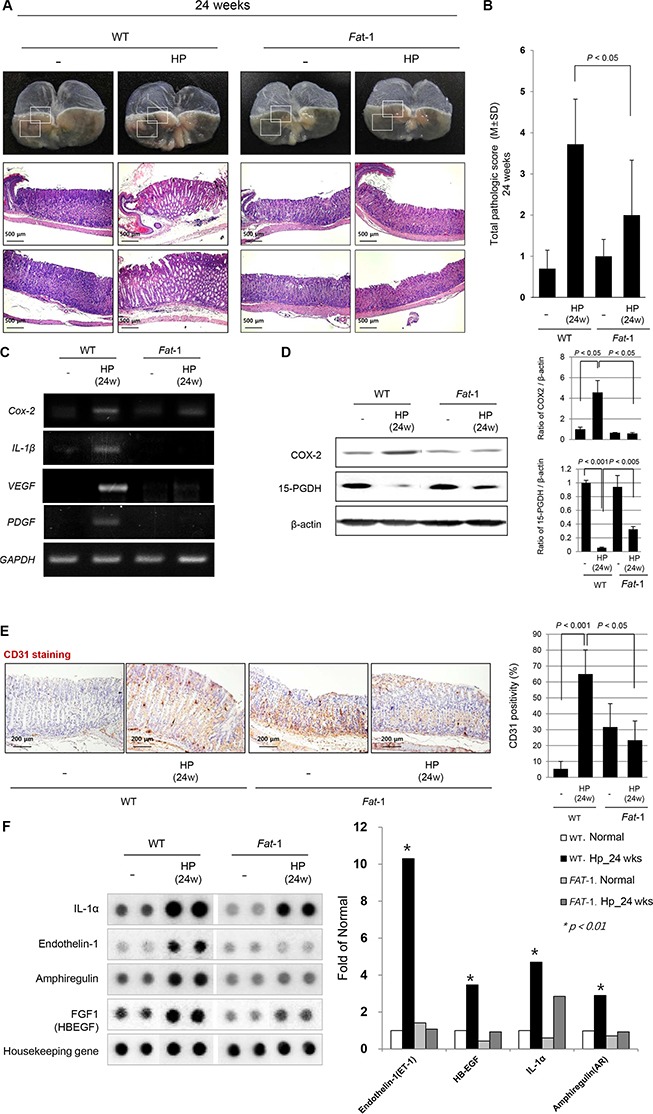
Mitigated chronic atrophic gastritis in *Fat*-1 TG mice compared to WT mice at 24 weeks after *H. pylori* infection (**A**) *H. pylori* infection in WT mice lead to significant gross changes at 24 weeks. Some portion of gastric mucosa looks like nodular and granular appearance, while the other portion of stomach looks thin and transparent. However, the gross lesions of *H. pylori*-infected *Fat*-1 TG mice were not changed compared to non-infected WT or *Fat*-1 TG mice even at 24 weeks. Almost all *H. pylori*-infected WT mice showed multiple nodular lesions accompanied with thin surrounding gastric wall, whereas no significant changes were noted in *Fat*-1 TG mice at 24 weeks (H&E stain, magnification, × 40, 100). (**B**) Pathological evaluation showed gastritis cystica profunda, disappearance of parietal cells, and profuse inflammatory cell infiltrations on submucosa and mucosa at *H. pylori*-infected WT mice. On the other hand, there were no prominent changes in *H. pylori*-infected *Fat*-1 TG mice, only showing mild gastritis and focal erosive changes. The pathological scoring between *H. pylori*-infected WT and *Fat*-1 TG mice were significantly differed at 24 weeks (*P* < 0.05). (**C**) Expressions of *Cox-2*, *IL-1*β, *VEGF*, and *PDGF* mRNA were significantly increased after *H. pylori* infection in WT mice, but much lesser in *Fat*-1 TG mice even after *H. pylori* infection at 24 weeks. (**D**) Western blot analysis for COX-2 and 15-PGDH. The expressions of COX-2 were significantly increased after *H. pylori* infected in WT mice group, but their expressions were significantly decreased in the *H. pylori-*infected *Fat*-1 TG mice group (*P* < 0.05). *H. pylori* infection in WT mice significantly cancelled the expressions of 15-PGDH at 24 weeks (*P* < 0.001), but 15-PGDH expressions were significantly preserved in *Fat*-1 TG mice, though decreased compared to non-infected group (*P* < 0.005). (**E**) The immunohistochemical changes of endothelial marker CD31 denoting angiogenic activity according to groups (Magnification, × 100). *H. pylori* infection led to significant increased positivity in WT mice (*P* < 0.001), but CD31 immunostaining was significantly attenuated in *Fat*-1 TG mice (*P* < 0.05). (**F**) Cytokine protein array. *H. pylori* infection in WT mice showed significantly increased expressions of IL-1α, endothelin-1, amphiregulin, FGF1 (*P* < 0.01), but never in *H. pylori*-infected *Fat*-1 TG mice, suggesting ischemic condition and proliferative activities were relived in *Fat*-1 TG mice even under *H. pylori* infection.

Contrary to gross lesions at 16 weeks, *H. pylori* infection lead to significant gross changes at 24 weeks, as seen in Figure 2A and Supplementary Figure S2; portions of the gastric mucosa had a nodular and granular appearance, while the remaining portion of stomach looked thin and transparent. Instead, the gross lesions of *H. pylori*-infected *Fat*-1 TG mice were not changed compared to non-infected WT or *Fat*-1 TG mice even at 24 weeks. Some mice administered only HSD without *H. pylori* infection showed some mass-like lesion on gross observation (see Supplementary Figure 2A), whereas none were observed in HSD-administered *Fat*-1 TG mice. Almost all *H. pylori*-infected WT mice showed multiple nodular lesions accompanied by a thin surrounding gastric wall, whereas no significant changes were noted in *Fat*-1 TG mice at 24weeks (see Supplementary Figure 2A and 2B). On pathological analysis, gastritis cysticaprofunda, disappearance of parietal cells, and profuse inflammatory cell infiltrations on submucosa and mucosa were noted at *H. pylori*-infected WT. As seen in Figure 2A, there were no prominent changes in *H. pylori*-infected *Fat*-1 TG mice, only mild gastritis and focal erosive changes were observed. The pathological scoring between *H. pylori*-infected WT and *Fat*-1 TG mice was differed significantly at 24 weeks (*P* < 0.05, Figure 2B). Since there were significant differences in inflammatory activities, proliferative status, and angiogenic activities between WT and *Fat*-1 TG mice at 24 weeks of *H. pylori* infection (Figure 2B), we had measured the expression levels of the inflammatory mediators *Cox-2* and *IL-1β* and angiogenic growth factors *VEGF* and Platelet-derived growth factor (PDGF); there were significant differences between WT and *Fat*-1 TG mice (Figure 2C). Further evaluation of COX-2 and Prostaglandin dehydrogenase (PDGH) was done by Western blots, COX-2 levels were significantly increased in WT mice after *H. pylori* infection (*P* < 0.05), but not in *Fat*-1 TG mice. The expression of 15-PGDH was significantly lower in *H. pylori*-infected WT mice at 24 weeks (*P* < 0.001*),* but significantly preserved at *Fat*-1 TG mice (*P* < 0.05, Figure 2D). In order to compare the angiogenic activities, we performed immunohistochemical staining with Cluster of differentiation (CD)31 endothelial antibody. As seen in Figure 2E, *H. pylori* infection led to a significant increase of CD31 in gastric mucosa (*P* < 0.001), but these expressions were not increased in *H. pylori*-infected *Fat*-1 TG mice. Though the expression of CD31 was higher in *Fat*-1 TG mice compared to WT, there was no statistical significance. In order to screen further changes in molecular events between groups, we performed protein array analysis. As seen in Figure 2F, *H. pylori* infection in WT mice led to significant increases in IL-1α, endothelin-1, amphiregulin, and Fibroblast growth factor (FGF) (*P* < 0.01), however, no significant changes were observed in *Fat*-1 TG mice, suggesting that the ischemic and proliferative conditions in *H. pylori*-infected WT mice were significantly relieved in *H. pylori*-infected *Fat*-1 TG mice.

### Increased *H. pylori*-induced tumorigeneis in WT mice, but not in *Fat*-1TG mice observed at 32 weeks

**Figure 3:**
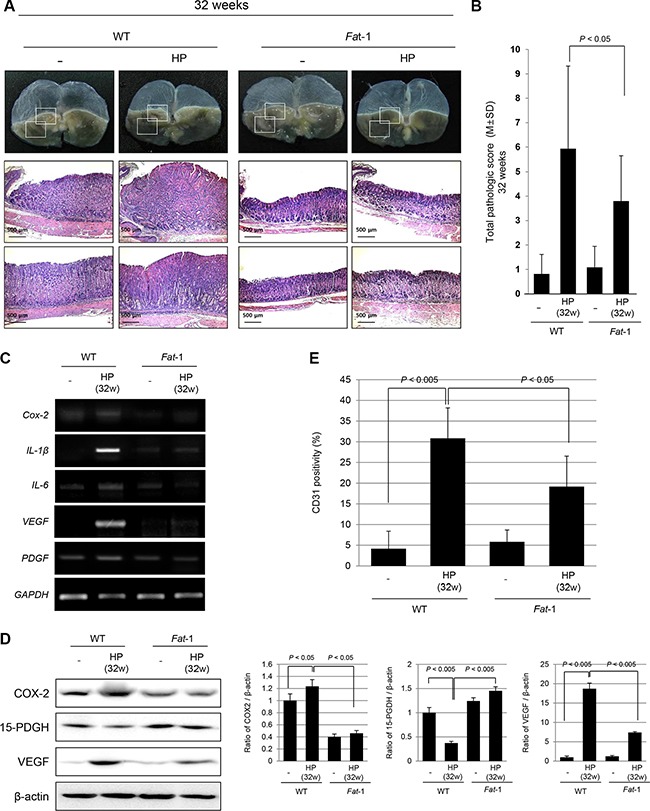
Significantly attenuated tumorigenesis in *Fat*-1 TG mice at 32 weeks of *H. pylori* infection (**A**) Current model of *H. pylori* infection followed with 7.5% high salt diet provoked significant tumorigenesis after 32 weeks of *H. pylori* infection on gross observation. There were multiple ulcerated tumors on the stomach accompanied with several nodules, but very thin gastric wall, transparently looking. However, no significant changes in gross appearance were noted in *H. pylori*-infected *Fat*-1 TG mice except small sized nodular lesions in 30% *Fat*-1 TG mice. Pathological findings in *H. pylori*-infected WT mice at 32 weeks showed gastric adenoma and chronic atrophic gastritis (H&E stain, Magnification, × 40). (**B**) Pathological scoring between groups showed significant differences in pathological scores between WT and *Fat*-1 TG mice (*P* < 0.05). (**C**–**D**) RT-PCR for inflammatory and angiogenic growth factors and immunohistochemical staining for CD31 were done. There were significant increases in *Cox-2*, *IL-1β*, *IL-6*, *VEGF*, *PDGF* mRNA and CD31 in *H. pylori*-infected WT mice, but significantly decreased in *Fat*-1 TG mice. (D) Western blots for COX-2, 15-PGDH, and VEGF according to group. The expressions of 15-PGDH were significantly decreased in *H. pylori*-infected WT mice (*P* < 0.005), while COX-2 expressions were significantly increased in WT mice at 32 weeks of *H. pylori* infection. On the other hand, the expressions of tumor suppressive 15-PGDH were significantly preserved in *H. pylori*-infected *Fat*-1 TG mice (*P* < 0.005). Just like COX-2, there were significant decrements in VEGF expressions in *Fat*-1 TG mice compared to WT mice.

Our model of *H. pylori* infection followed with 7.5% high salt diet provoked significant tumorigenesis after 32 weeks of *H. pylori* infection on gross observation (see Supplementary Figure S3A). As seen in Figure 3A, there were multiple ulcerated tumors on the stomach accompanied with several nodules, but very thin stomach walls at 32 weeks, whereas still no significant changes in gross appearance were noted in *H. pylori*-infected *Fat*-1 TG mice except that small sized nodular lesions were noted in 30% *Fat*-1 TG mice (see Supplementary Figure S4A and S4B). On pathological findings, gastric adenoma and chronic atrophic gastritis was prominent pathology observed at WT mice. On pathological scoring between groups, significant differences in pathological scores were seen at 32 weeks after *H. pylori* infection (Figure 3B). In order to elucidate the underlying molecular changes of these inflammatory and tumorigenic changes between groups, we performed RT-PCR to quantify the levels of inflammatory and angiogenic growth factors including *Cox-2, IL-1β, IL-6, VEGF, and PDGF* mRNA (Figure 3C) and immunohistochemical staining for CD31 (Figure 3E). As noted at 24 weeks, there were significant increases in the mRNA levels of *Cox-2*, *IL-1β*, *IL-6*, *VEGF*, *PDGF* and expressions of CD31 in *H. pylori*-infected WT mice, however no changes in *Fat*-1 TG mice at 32 weeks. The expression level of 15-PGDH was significantly decreased in *H. pylori*-infected WT mice (*P* < 0.005), and significantly preserved in *Fat*-1 TG mice. The expression levels of COX-2 and VEGF were similar, there were significant decreases in the oncogene COX-2 and VEGF, and significant increases in the expression of the tumor suppressive 15-PGDH in *Fat*-1 TG mice compared to WT mice (Figure 3D).

### Long-term (45 weeks) preventive effects of ω-3 PUFA-producing *Fat*-1 TG mice against *H. pylori*-induced gastric carcinogenesis

**Figure 4:**
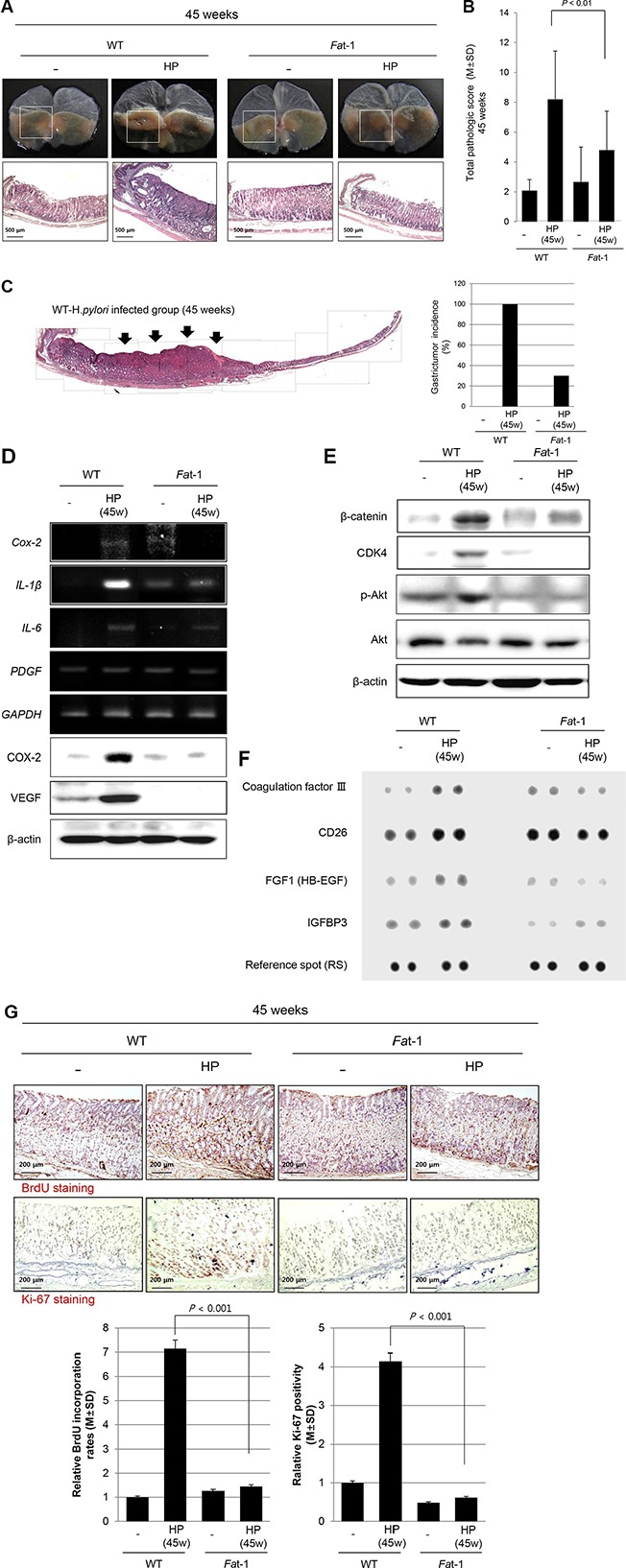
Long-term efficacy of ω-3 PUFA on *H. pylori*-induced gastric tumorigenesis at 45 weeks (**A**) All of *H. pylori*-infected mice had developed variable sized gastric tumors, whereas only 30% mice developed gastric tumors in *Fat*-1 TG mice, smaller in size than WT mice (H&E stain, Magnification, × 40). (**B**–**C**) The mean pathological scoring was significantly decreased in *Fat*-1 TG mice compared to WT mice (*P* < 0.01). 1:1 mounting view of whole stomach of WT - *H. pylori* group showed moderately-differentiated adenocarcinoma, by which there was significant difference in tumorigenesis between WT and *Fat*-1 TG mice at 45 weeks after *H. pylori* infection. (**D**) Measuring the expressions of *Cox-2*, *IL-1β*, *IL-6*, and *PDGF* mRNA between groups, *H. pylori*-infected WT showed significantly increased expressions, but significantly attenuated at *Fat*-1 TG mice. (**E**) Western blots for β-catenin, CDK4, phosphorylated-Akt, and total Akt according to group. (**F**) Protein array. (**G**) Immunohistochemical staining for BrdU and ki-67. All of figure E, F, and G consistently suggested *H. pylori* infection for 45 weeks led to significantly increased proliferative actions in WT mice, but these mucosal proliferative mechanisms were significantly decreased in *Fat*-1 TG mice (*P* < 0.001, Magnification × 100). The stomachs were stained for BrdU and Ki67, the number of positive cells was represented as mean ± SD.

We maintained our model up to 45 weeks after *H. pylori* infection to compare the chemo-preventive effects of ω-3 PUFAs. As seen in Figure 4A and Supplementary Figure S4A, all *H. pylori*-infected mice had developed variable sized gastric tumors, whereas only 30% of *Fat*-1 TG mice developed gastric tumors and those were smaller than those in WT mice (see Supplementary Figure S4B). When reviewing the gross and pathological appearance, some tumors were shown to be more invasive and larger in size in WT mice compared to *Fat*-1 TG mice (Figure 4A and 4B). As seen in Figure 4C, 1:1 mounting view of whole stomach of WT – *H. pylori* group showed moderately-differentiated adenocarcinoma, by which there was significant difference in tumorigenesis between WT and *Fat*-1 TG mice at 45 weeks after *H. pylori* infection. The expression of important inflammatory and angiogenic growth factors, including *Cox-2*, *IL-1β*, *IL-6*, and *PDGF* mRNA are presented in Figure 4D and were significantly increased in *H. pylori*-infected WT, but not *Fat*-1 TG mice at 45 weeks. The protein expressions of COX-2 and VEGF were noted in similar way. Explaining the significant differences in tumorigenesis between groups, proliferative activities were measured. β-catenin, Cyclin-dependent kinase (CDK)4, phosphorylated Protein kinase B (PKB or AKT) (Figure 4E), CD26, Heparin-binding EGF-like growth factor (HB-EGF), Insulin-like growth factor-binding protein (IGFBP)3 (Figure 4F), Bromodeoxyuridine (BrdU), and Ki-67 (Figure 4G) were included in these measurements reflecting *H. pylori*-associated proliferative activities. As seen in Figure 4E, 4F, and 4G, *H. pylori* infection was associated with significant increases in β-catenin, CDK4, Akt activation, Ki-67 as well as BrdU incorporation, whereas these proliferative markers were not significantly different from the non-infected *Fat*-1 TG mice (Figure 4E and 4G). At this stage, we also conducted protein arrays to identify proteins whose expressiosn are significantly different between WT and *Fat*-1 TG mice. As seen in Figure 4F, CD26, FGF1 (HB-EGF), and IGFBP3 were significantly increased in *H. pylori*-infected WT, but not changed in *Fat*-1 TG mice.

### Dose of exogenous ω-3 PUFAs showing similar lipid profiles of stomach as seen in *Fat*-1 TG mice

**Figure 5:**
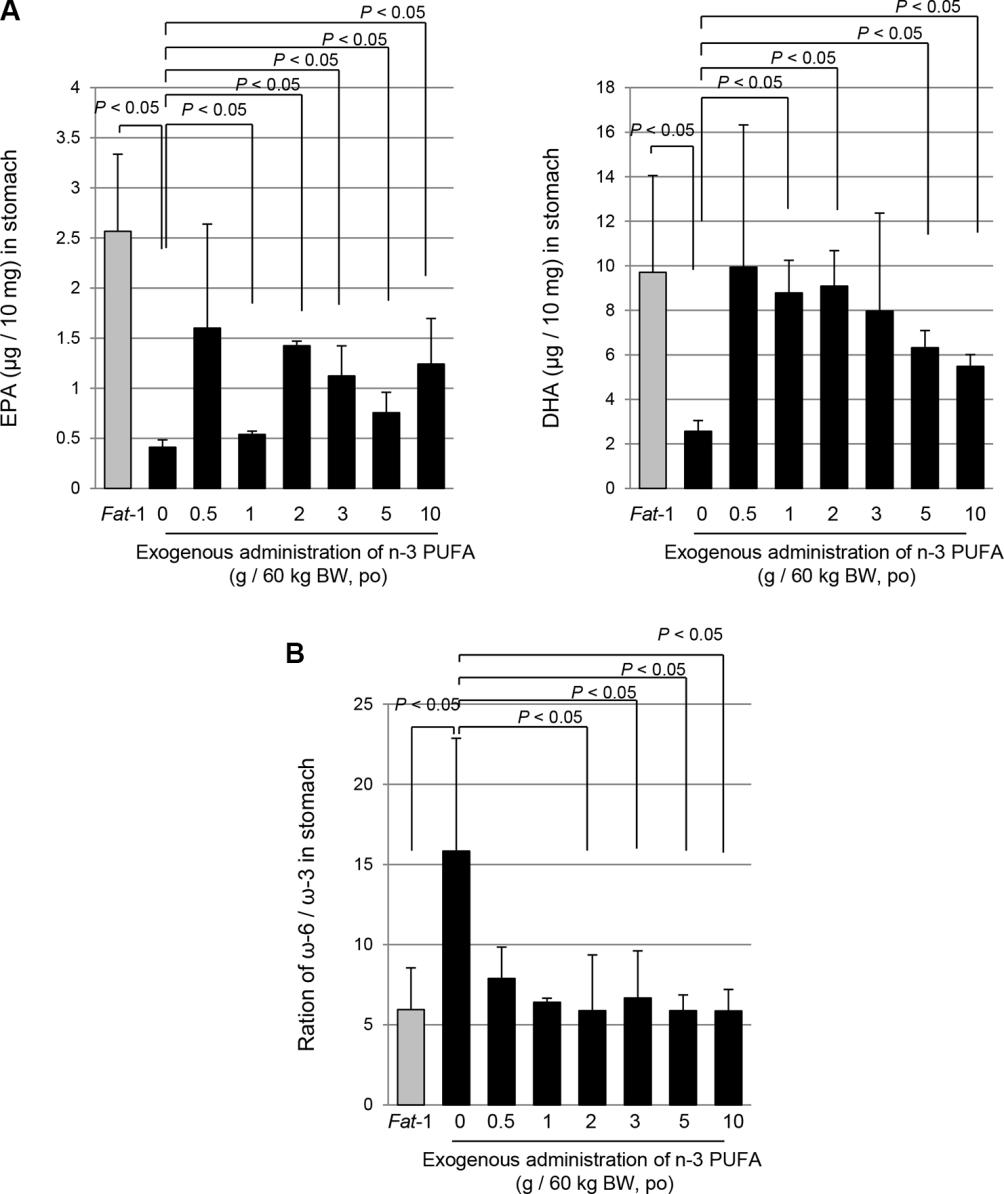
The comparison of concentration of DHA, EPA and ratio of ω-6 and ω-3 PUFAs; between WT mice following administration of exogenous ω-3 PUFA and endogenous ω-3 generated within *Fat*-1 TG mice (**A**–**B**) The DHA and EPA were extracted from stomach of *Fat*-1 TG mice and WT mice administrated with ω-3 dose dependently and analyzed by LC/MS/MS as described in Materials and Methods.

Though the current experiment investigated the exact role of ω-3 PUFAs against *H. pylori* infection by using *Fat*-1 TG mice generating ω-3 PUFAs in the stomach after being fed ω-6 PUFAs rich diets, we tested how much exogenous ω-3 PUFAs is required to achieve the above protection from *H. pylori* infection. We administered various doses of ω-3 PUFAs (0.5 g/60 kg to 10 g/60 kg) through oral feeding and compared the eicosapentaenoic acid (EPA) and docosahexaenoic acid (DHA) levels in the stomach. As seen in Figure 5A and 5B, ω-3 PUFAs more than 0.5 g/60 kg showed similar patterns in *Fat*-1 TG mice because the ratio of ω-6 PUFAs/ω-3 PUFAs was noted in mice fed more than 0.5 g/60 kg ω-3 PUFAs (Figure 5B).

## DISCUSSION

In the current experiment, ω-3PUFAs attenuated *H. pylori*-associated gastric inflammation, rejuvenated *H. pylori*-induced CAG, and prevented *H. pylori*-associated gastric tumorigenesis in *Fat-*1 TG mice. In terms of cancer prevention, concerted mechanisms including COX-2 inhibition, 15-PGDH preservation, regulation of proliferation, and mitigated tumorigenic angiogenesis were revealed. Through measuring real levels of lipid profiles in the stomach after exogenous administration of ω-3 PUFAs, we found that dietary intake of more than 0.5 g/60 kg ω-PUFAs could achieve similar lipid profile as seen in *Fat*-1 TG mice. To our knowledge, this might be the first study demonstrating the inhibitory effect of ω-3PUFAs on *H. pylori*-induced inflammation and gastric tumorigenesis, signifying the future potential of ω-3 PUFAs to ameliorate either *H. pylori*-associated CAG or gastric cancer through dietary intervention in the clinic.

Under the same *H. pylori* infection conditions, the expression of inflammatory and angiogenic growth factors were significantly reduced in *Fat*-1 TG mice compared to WT (Figure 1D). This suggests that ω-3 PUFAs were capable of suppressing the main inflammatory mechanism and providing an efficient gastro-protection against *H. pylori* infection beyond inhibitory actions on *H. pylori* colonization. However, since several *in vitro* and *in vivo* studies showed that ω-3 PUFAs could inhibit *H. pylori* growth *in vitro* and its colonization in the gastric mucosa of mice *in vivo* [22, 23], we have investigated the possibility of inferior colonization or bactericidal effects due to ω-3 PUFAs synthesized in *Fat*-1 TG mice. Similar colonization statuses as measured using direct culture, rapid urease test, *e.g.,* CLO test, and Giemsa staining were observed in WT and TG mice up to 8 weeks of colonization. To avoid reduced bacterial colonization in *Fat*-1 TG mice, we administered the proton pump inhibitor, pantoprazole, before *H. pylori* inoculation to enhance their colonization rates followed with thrice administration of *H. pylori* cultures, and following this protocol, we did not find any differences in *H. pylori* colonization between WT and *Fat*-1 TG mice. Therefore, we could rule out that the cancer prevention outcome was not related with lowered colonization in *Fat*-1 TG mice, even though an *in vitro* study showed some bacterostatic effects of EPA or DHA.

To determine the molecular mechanisms of the ω-3PUFAs-mediated decrease in *H. pylori*-associated inflammation 16 to 24 weeks after *H. pylori* infection, we first looked at inflammatory mediators and angiogenic growth factors. Since the increased expression of inflammatory mediators such as COX-2, IL-1β, IL-6, IFN-γ, and IL-8 were one of core pathogenic mechanisms in *H. pylori*-associated gastritis [24, 25] and these inflammatory responses are thought to be one of the core processes involved in gastric carcinogenesis [26, 27], we have focused on to the serial changes of COX-2-Prostaglandin E2 (PGE2) pathway [28]. As seen in Figures 1, 2, and 3, these inflammatory mediators were significantly decreased in *Fat*-1 TG mice compared WT mice. Both ω-6 and ω-3 PUFAs are precursors of potent lipid mediators termed eicosanoids, eicosanoids derived from ω-6 PUFAs have pro-inflammatory and immune-active functions, whereas eicosanoids derived from ω-3 PUFAs have anti-inflammatory properties [13, 29]. This is why the increased intake of ω-3 PUFAs led to a decrease in the risk of many chronic inflammation-based diseases including arthritis, diabetes, obesity, cardiovascular diseases, inflammation, and cancer [30, 31]. COX can generate anti-inflammatory mediators from ω-3 PUFAs [32] including: i) eletrophilicoxo-derivatives (EFOX), a peroxisome proliferator-activated receptor-γ (PPAR-γ) agonist that mostly transduces the beneficial anti-inflammatory effects of DHA, ii) COX inhibitor, iii) aspirin and iv) ω-3 PUFAs-derived lipid autacoids termed resolvins and protectins [33]. Similarly, even though we have published the cancer-preventive effects of the non-steroidal anti-inflammatory drugs (NSAIDs) nimesulide and celecoxib [34] and multiple case-control cohort randomized control trial (RCT), a pooled analysis, and meta-analyses also suggest a preventive effect of aspirin or NSAIDs on the development of non-cardia gastric cancer [35]. The adverse effects associated with the use of NSAIDs may lead to poor adherence as chemo-preventive agents and lead to other harmful effects on the kidney and cardiovascular system. Therefore, it should be emphasized that ω-3 PUFAs raises low toxicological constraints.

Secondarily, the observation that at 24 weeks, the preservation of 15-PGDH—a PG degrading enzyme—accompanied with attenuated COX-2, VEGF, PDGF, and CD31 expression in *Fat*-1 TG mice compared to WT mice strongly explained the cancer-preventive actions of ω-3 PUFAs [36]. Since 15-PGDH may function as a tumor suppressor through antagonizing oncogenic action of COX-2, 15-PGDH has been found to be down-regulated and a contributor to elevated levels of PGE2 in most tumors, as seen in our *H. pylori*-associated gastric carcinogenesis model. Since the expression of 15-PGDH and COX-2 appears to be regulated reciprocally in cancer cells, up-regulation of 15-PGDH can be either down-regulated by transcriptional repressors or the attenuation of enzymatic turnover [37]. From our study, significant down-regulation was noted in WT mice after *H. pylori* infection, but preserved or elevated expression levels of 15-PGDH were observed in *Fat*-1 TG mice. We documented that ω-3 PUFAs significantly up-regulated 15-PGDH to prevent tumorigenesis relevant to intestinal polyposis [38]. Regarding the changes of decreased 15-PGDH following *H. pylori* infection [39], the decreased expression of 15-PGDH were reversed with successful *H. pylori* eradication, in which suppressed 15-PGDH expression levels were associated with Toll like receptor-4 and Myeloid differentiation primary response gene 88 expressions, phospho-Extracellular signal-regulated kinases1/2, and EGF receptor (EGFR)-Snail [40].

Thirdly, considering findings from 32 to 45 weeks in *Fat*-1 TG mice after *H. pylori* infection (Figure 3 and Figure 4), co-mechanisms regulating abnormal mucosal growth and considerable rejuvenation were orchestrated to prevent *H. pylori*-gastric carcinogenesis. As much as the above mentioned tumor suppressive 15-PGDH and sustained inhibition of oncogenic inflammatory mediators, significant control of abnormal mucosal proliferation was observed in *Fat*-1 TG mice. The proliferation of raised cells is a typical observation in either pre-neoplasia or neoplasia because cells in the proliferative stage are vulnerable to somatic mutations, especially frequently observed in inflammation-prevailed microenvironment conditions. Among the well-known mechanisms of *H. pylori* promoted gastric carcinogenesis, are abnormal mucosal proliferation [41, 42], promoted epithelial mesenchymal transition [43] via abnormal Akt or Wnt/β-catenin or sonic hedgehog oncogenic activation [44–46], aberrant promoter methylation in tumor suppressor genes, abnormal expression of microRNAs, and abnormal apoptosis [47] after the stage of CAG. As seen in Figure 2F, the expression of IL-1α, amphiregulin, and FGF1 were significantly increased after 24 weeks of *H. pylori* infection and the expression of β-catenin, CDK4, phosphorylated-Akt, CD26, coagulation factor III, IGFBP3 were significantly increased after 45 weeks of *H. pylori* infection. Among these factors, CD26, a surface dipeptidylpeptidase IV—a multifunctional protein with intrinsic peptidase activity that inactivates or degrades some bioactive peptides [48]. Due to its multiple functions, CD26/Dipeptidyl peptidase 4 has been shown to be related to the tumor-development process, but it remains unclear if it is a tumor suppressor or a marker of malignancy. However, all of these expressions relevant to abnormal mucosal growth were all mitigated in *Fat-1* TG mice. Significantly, we observed that some of these biomarkers were already used for cancer risk of *H. pylori*-associated gastric cancer except for CD26 [49–53]; ω-3 PUFAs can be an effective agent to counteract biomarkers suggestive of cancer risk.

Fourthly, although they were not studied in this study, additional mechanisms of ω-3 PUFAs-mediated chemoprevention include its role as a structural component providing the optimal function of cellular membranes including: i) membrane fluidity, ii) enzyme activity, iii) balanced ω-6:ω-3 PUFAs production and gene expressions, suggesting that increasing the amount of ω-3 PUFAs consumed at the population level may be a possible potential health benefit; increased intake of ω-3 PUFAs result in prevention of chronic diseases and reduction in the burdening of the health care. From our study, we also investigated why a number of ω-3 PUFAs–containing dietary supplements have shown on the market claiming to protect against the development of a variety of conditions including cancer, but meta-analysis did not provide results of evidence based medicine to suggest a significant association between ω-3 PUFAs and cancer incidence [54]. In the current study, we have compared the gastric levels of ω-3 PUFAs after administering several dose of ω-3 PUFAs (Figure 5) and found that intake more than 0.5 g/60 kg resulted in a similar ω-6/ω-3 PUFAs ratio in the stomach. Our results support the use of dietary ω-3PUFAs in *H. pylori*-infected patients as a safe prophylactic/preventive strategy before stepping into irreversible condition. Clinical trials to document the chemo-preventive effects of ω-3 PUFAs have mostly focused on colorectal polyps; Higurashi T *et al.* [55] performed a double blind, placebo-controlled RCT to explore the effects of EPA against colorectal aberrant crypt foci (ACF) and highlighted the suppressive effect of 2.7g EPA/day on the formation of ACF and Hull MA *et al.* [56], after their SeAFOod trials, The seafood Polyp Prevention Trial, concluded that EPA can prevent polyp formation.

Lastly, we have checked microbiota changes in WT and *Fat*-1 TG mice during 24 and 32 weeks through 454 pyrosequencing measurement and found significant changes between WT and *Fat*-1 TG mice (data not shown). These significant changes in fecal microbiota in *Fat*-1 TG mice might come from the following two possibilities: i) the direct influence of ω-3 PUFAs on microbiota change or ii) changes reflecting mitigated conditions of *H. pylori*-induced chronic atrophic gastritis by fatty acid produced in *Fat-*1 TG mice. Conclusively, our findings suggest that he increased abundance of ω-3PUFAs in tissue significantly reduced gastric inflammation and tumorigenesis in *Fat*-1 mice. Clinical trials to document the effects of exogenous administration of more than 0.5 g/60 kg high purity ω-3 PUFAs in chronic *H. pylori* infection should be conducted to determine the benefit of the use of ω-3 PUFAs in chemo-preventive strategies for *H. pylori*-induced atrophic gastric disease.

## MATERIALS AND METHODS

### *H. pylori*-infected mice model

#### Animals

Five-week-old male C57BL/6 mice (WT mice) were purchased from Orient (Seoul, Korea) and *Fat*-1 transgenic (*Fat*-1 TG) mice were provided kindly from Dr. Jing X. Kang (Boston, MA). They were housed in a cage maintained at 23°C in a 12 h/12 h light/dark cycle under specific pathogen-free conditions (*n* = 160). We divided four groups; 1) WT mice as vehicle control group 2) WT mice as *H. pylori*-infected group 3) *Fat*-1 mice as vehicle control group 4) *Fat*-1 mice as *H. pylori*-infected group. First, all groups were given intraperitoneal (*i.p*.) injections of proton pump inhibitors (PPIs, pantoprazole, 20 mg/kg; Amore-Pacific Pharma) three times per week to facilitate *H. pylori* colonization through lowered gastric acidity. And then, each mouse was intragastrically inoculated with a suspension of *H. pylori* containing 10^9^ CFUs/mL or with an equal volume (0.1 mL) of clean TS broth using gastric intubation needles. The *H. pylori*-infected mice were fed a special pellet diet based on AIN-46A containing 7.5% NaCl (high salt diet, Biogenomics, Seongnam, Korea) for 45 weeks (Figure 1A) to promote *H. pylori*-induced carcinogenic process in all infected animals [57]. And randomized groups of mice (*n* = 10) sacrificed at 16, 24, 32 and 45 weeks post *H. pylori* infection, respectively. The stomachs of mice were opened along the greater curvature and washed with ice cold PBS. The numbers of either erosions/ulcers or protruded nodule/mass were determined under the magnified photographs (see Supplementary Figures S1–S4). Stomachs were isolated and subjected to a histologic examination, ELISA, Western blotting, and RT-PCR. All animal studies were carried out in accordance with protocols approved by the Institutional Animal Care and Use Committee (IACUC) of CHA University CHA Cancer Institute after IRB approval.

#### Gross lesion index

After sacrificing the mice, the isolated stomachs were open along the greater curvature and washed in ice-cold saline. To investigate the degree of gross mucosal pathology, the mucosal sides of the stomachs were photographed using a digital camera and part of the mucosa was immediately fixed with 10% formalin solution. The gross damage of the gastric mucosa was assessed by three gastroenterologists, who were blinded to the treatments, using a gross ulcer index [58]. All the gross photographs were displayed in Supplementary Figures and tumorous lesion was depicted with white arrow.

#### Index of histopathologic injury

For histopathological analysis, the stomach were fixed in 10% neutralized buffered formalin, processing using the standard method and embedded in paraffin. Sections of 4 μm thickness were then stained with hematoxylin and eosin [59]. The glandular mucosae of corpus and antrum were examined histologically. The pathological changes of *H. pylori*-infection, such as inflammatory cells infiltration, erosive lesions, ulceration, dysplasia, adenoma formation (precancerous lesion), were graded by three gastroenterologists, who were blinded to the group, using an index of histologic injury defined [60]. In this study, inflammation was defined as grade the infiltration of inflammatory cells, 0: none, 1: under the lamina propria, 2: half of mucosa 3: until the epithelial gland layer (all mucosa). The erosion was defined as proportion of erosive lesion, 0: none, 1: loss of epithelial gland layer (1/3 proportion), 2: two-three portion of mucosa (2/3 proportion) 3: all mucosa (3/3 proportion)

#### BrdU staining for assessing mucosal proliferation

To estimate the rate of proliferation that is increased during carcinogenesis, we injected BrdU before sacrifice, and performed immunohistochemical analysis with anti-BrdU antibody.

#### Immunohistochemical staining

Immunohistochemistry was performed on replicate sections of mouse gastric tissues. After deparaffinization were dewaxed and rehydrated with graded alcohol, and boiled three times in 100 mM Tris buffered saline (pH 6) with 5% urea in an 850 W microwave oven for 5 min each. And then cooling in water for 15 min and washed in PBS, and slides were incubated overnight with the primary antibody at 4°C. Antibodies: F4/80 (1:500; eBioscience, San Diego, CA) or CD31 (1:300; Dako, Santa Clara, CA) or Ki-67 (1:300; Santa cruz, Santa Cruz, CA) in the presence of 1.0% bovine serum albumin respectively. Slides incubated with secondary antibody (1:300) for 1 h at room temperature, and then with 40-6- diamidino-2-phenylindole (DAPI, 100 ng/ml) for 1 min at room temperature. And finally the slides were counterstained with hematoxylin (Sigma-Aldrich).

#### RT-PCR

Total RNA was isolated using the Trizol (Invitrogen, Carlsbad, CA). Trizol was added to 1.5 ml tube, which were then incubated 10 min at 4°C and gently mixed with 100 μl chloroform (Merck, Rahway, NJ). After incubation for 10 min in ice, samples were centrifuged at 10,000 *g* for 30 min. Supernatants were extracted and mixed with 200 μl isopropanol (Merck), and mixtures were incubated at 4°C for 1 h. After centrifuging at 13,000 *g* for 30 min, pellets were washed with 70% (*v*/*v*) ethanol. After allowing the ethanol to evaporate completely, pellets were dissolved in 40 μl diethylene pyrocarbonate-treated water (Invitrogen Life Technologies). cDNA was prepared using reverse transcriptase originating from Murine-Moloney leukemia virus (Promega, Madison, WI), according to the manufacturer's instructions. The polymerase chain reaction (PCR) was performed over 25 cycles of: 94°C for 20 s, 58.5 for 30 s, and 72°C for 45 s. Oligonucleotide primers were purchased from Bioneer (Daejeon, Korea). Oligonucleotide primers were as follows; for VEGF, sense 5′-GAA GCT ACT GCC GTC CGA TT-3′ and antisense 5′-TCC TCT TCC TTC ATG TCA GGC-3′, for COX-2, sense 5′-GAA ATG GCT GCA GAG TTG AA-3′ and antisense 5′-TCA TCT AGT CTG GAG TGG GA-3′, for PDGF, sense 5′-ACG TCA TGT TAC GGC TTC CT-3′ and antisense 5′-CAG TGT GAC TGT GTC TCC CC-3′, for IL-1β, sense 5′-CAG GCT CCG AGA TGA ACA ACA AAA-3′ and antisense 5′-TGG GGA ACT CTG CAG ACT CAA ACT-3′, for IL-8, sense 5′-GGG GCT TTG CCG TGC AAT AA-3′ and antisense 5′-GCA CAG GGT TGA GCC AAA A-3′, for IFN-γ, sense 5′-ACA ATG AAC GCT ACA CAC TG-3′ and antisense 5′-TCA AAC TTG GCA ATA CTC AT-3′, for IL-6, sense 5′-AAG AGA CTT CCA GCC AGT TG-3′ and antisense 5′-TGG ATG GTC TTG GTC CTT AG-3′, and for GAPDH, sense 5′-GGT GCT GAG TAT GTC GTG GA-3′ and antisense 5′-TTC AGC TCT GGG ATG ACC TT-3′.

#### Western blotting

Cells were harvested and lysed in lysis buffer (Cell signaling Technology) containing 1 mM phenylmethylsulfonyl fluoride (PMSF, Sigma Aldrich St. Louis, MO). After 30 min of incubation, samples were centrifuged at 12,000 g for 15 min 4°C. The supernatants were then collected and protein quantification was carried out with a Bio-Rad protein assay. Equal amounts soluble protein (30 μg) were denaturated by heating at 100°C for 3 minutes. Proteins were separated by sodium dodecyl sulphate-polyacrylamide gel electrophoresis (SDS-PAGE) and transferred to polyvinylidene fluoride membranes. The membranes were blocked in 5% BSA in PBST for 30 min. And then, the membranes probed initially with specific primary antibody, washed, incubated with peroxidase-conjugated secondary antibodies, and rewashed. The protein bands were detected by chemiluminescence (Supersignal, Pierce) exposure on chemiluminescence system (GE Healthcare, Buckinghamshire, UK). The general procedure for Western blot analysis of cultured mouse gastric mucosal cells was similar to the procedures described above. Antibodies used in the current study were COX-2, purchased from Thermo and β-actin, VEGF, β-catenin, purchased from Santa Cruz and CDK4, phospho-Akt, Akt, purchased from cell Signaling Technology and 15-PGDH, purchased from Cayman.

#### Cytokine protein array

Cytokine protein array was performed using Mouse Cytokine Antibody Array 3 (4 membrane arrays) with Accessories, for simultaneous detection of 54 cytokines related proteins in 2 samples from R&D systems (Minneapolis, MN). After blocking the array membranes for 30 minutes, the membranes were incubated with 1 ml of serum at room temperature for 2 hours. After washing with buffer, we added primary biotin-conjugated antibody to each membrane, for incubation at room temperature for 2 hours. After washing with buffer and addition of horseradish peroxidase-conjugated streptavidin to each membrane, we exposed them to detection buffer, using a luminescent image analyzer system (LAS-4000, Fuji Film; Tokyo, Japan). Density was expressed as the percentage of the detected value from the sample versus the background result, using a gelpro32 program (Media Cybernetics, Rockville, MD).

#### Gas chromatography for measuring lipid profiles in the stomach

Fatty acid profiles were analyzed using gas chromatography as described previously [19]. Briefly, 1 cm of mice tails (in order to perform the phenotyping of mice) or blocks of colon tissue (5 × 5 mm) were grounded to powder under liquid nitrogen. Samples were then subjected to extraction of total lipids and fatty acid methylation by heating at 100°C for 1 h under 14% boron trifluoride (BF3)–methanol reagent (Sigma, St. Louis, MO) and hexane (Sigma). Fatty acid methyl esters were analysed by gas chromatography using a fully automated 6890 N Network GC System (Agilent Technologies) equipped with a flame-ionization detector. Peaks of resolved fatty acids were identified by comparison with fatty acid standards (Nu-chek-Prep), and area percentage for all resolved peaks was analysed using GC ChemStation Software (Agilent Technologies, Santa Clara, CA) [61].

#### Statistical analyses

The data are presented as means ± standard deviations (S.D.). The data were analyzed by 1-WAY ANOVA, and the statistical significance between groups was determined by Student *t* test. Statistical significance was accepted at *P* < 0.05.

## SUPPLEMENTARY MATERIAL FIGURES



## Abbreviations

H. pylori: Helicobactor pylori
ω-3 PUFAs: Omega-3-polyunsaturated-fatty acids
WT: Wild-type
TG: Transgenic
COX: Cyclooxygenase
PGDH: Prostaglandin dehydrogenase
15-PGDH: Hydroxyprostaglandin dehydrogenase
MGC: Metachronous gastric cancer
IL: Interleukin
IFN: Interferon
VEGF: Vascular endothelial growth factor
PDGF: Platelet-derived growth factor
CD: Cluster of differentiation
FGF: Fibroblast growth factor
CDK: Cyclin-dependent kinase
AKT: Protein kinase B
HB-EGF: Heparin-binding EGF-like growth factor
IGFBP: Insulin-like growth factor-binding protein
BrdU: Bromodeoxyuridine
PGE2: Prostaglandin E2
RCT: Randomized control trial
