# Supplementary material for: Suppressed Helicobacter pylori-associated gastric tumorigenesis in Fat-1 transgenic mice producing endogenous ω-3 polyunsaturated fatty acids
Source: Oncotarget. 2016 Aug 12;7(41):66606–22. doi: 10.18632/oncotarget.11261 (PMC5341824; doi:10.18632/oncotarget.11261)
Supplement: Supplementary file 1 [file oncotarget-07-66606-s001.pdf]

Suppressed *Helicobacter pylori*-associated gastric tumorigenesis in Fat-1 transgenic mice producing endogenous ω-3 polyunsaturated fatty acids

Supplementary Materials

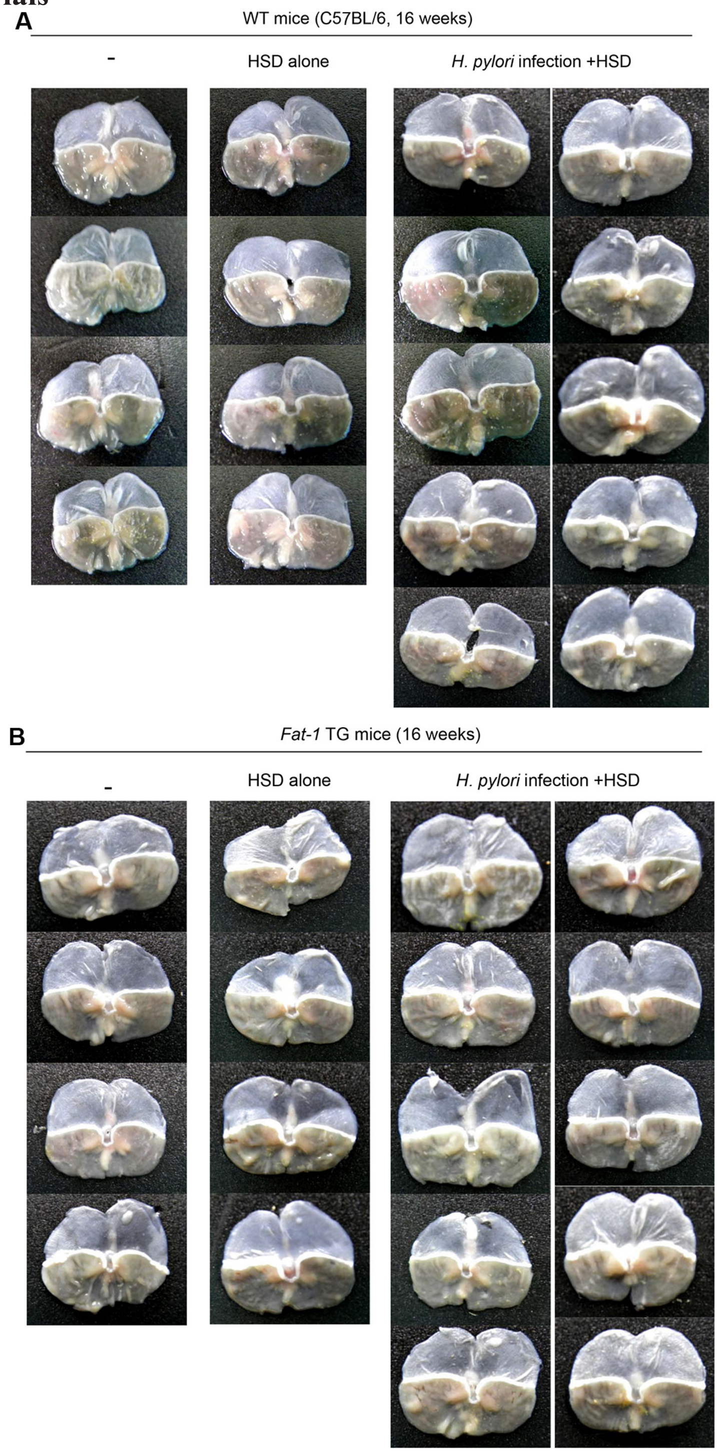

Supplementary Figure S1: Gross photographs of mice stomach according to group observed at 16 weeks. (**A**) Non-infected vehicle control group, HSD alone, and H. *pylori* infection of 16 weeks WT mice. (**B**) Non-infected vehicle control group, HSD alone, and H. *pylori* infection of 16 weeks *Fat-1* TG mice.

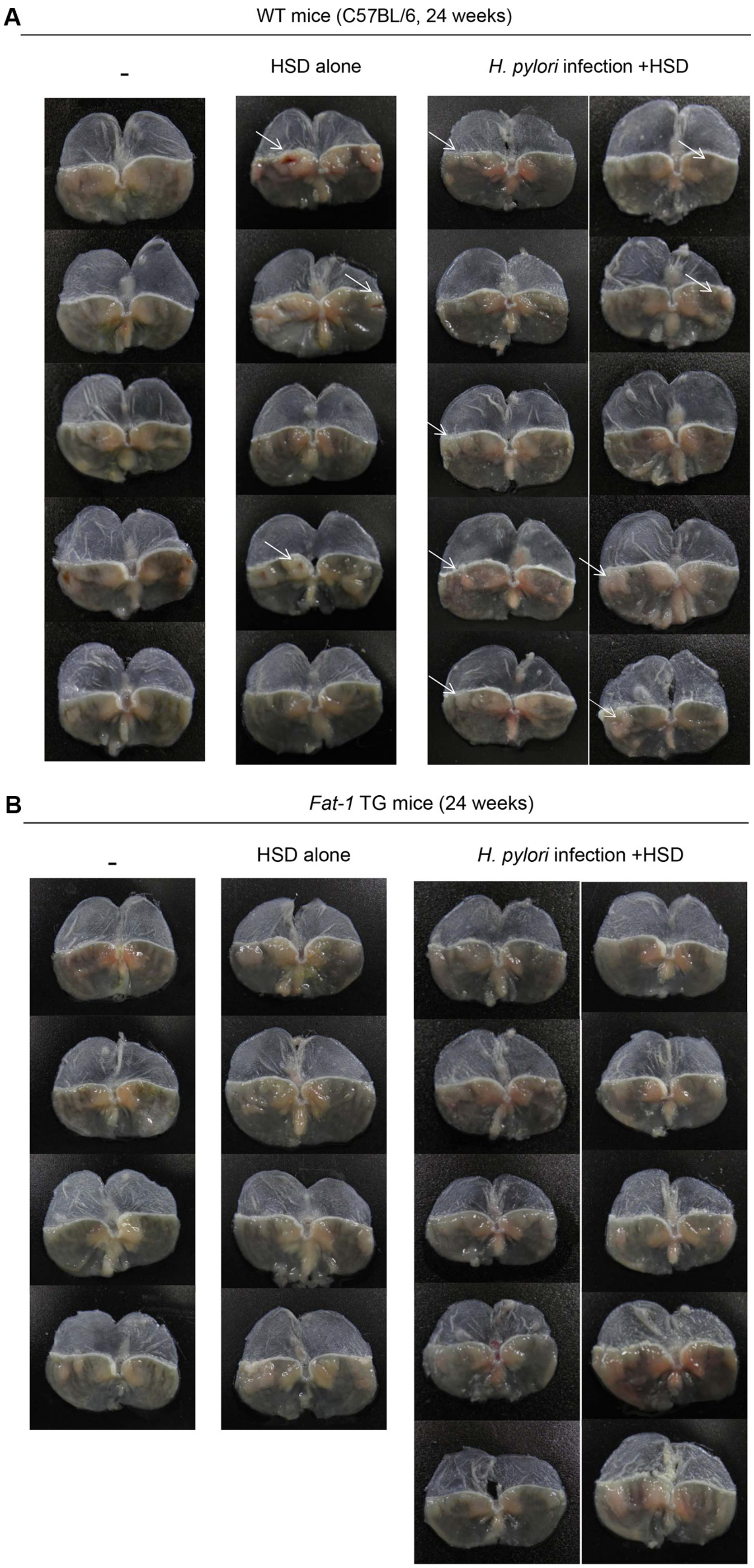

Supplementary Figure S2: Gross photographs of mice stomach according to group observed at 24 weeks. (**A**) Non-infected vehicle control group, HSD alone, and H. *pylori* infection of 24 weeks WT mice. (**B**) Non-infected vehicle control group, HSD alone, and H. *pylori* infection of 24 weeks *Fat-1* TG mice.

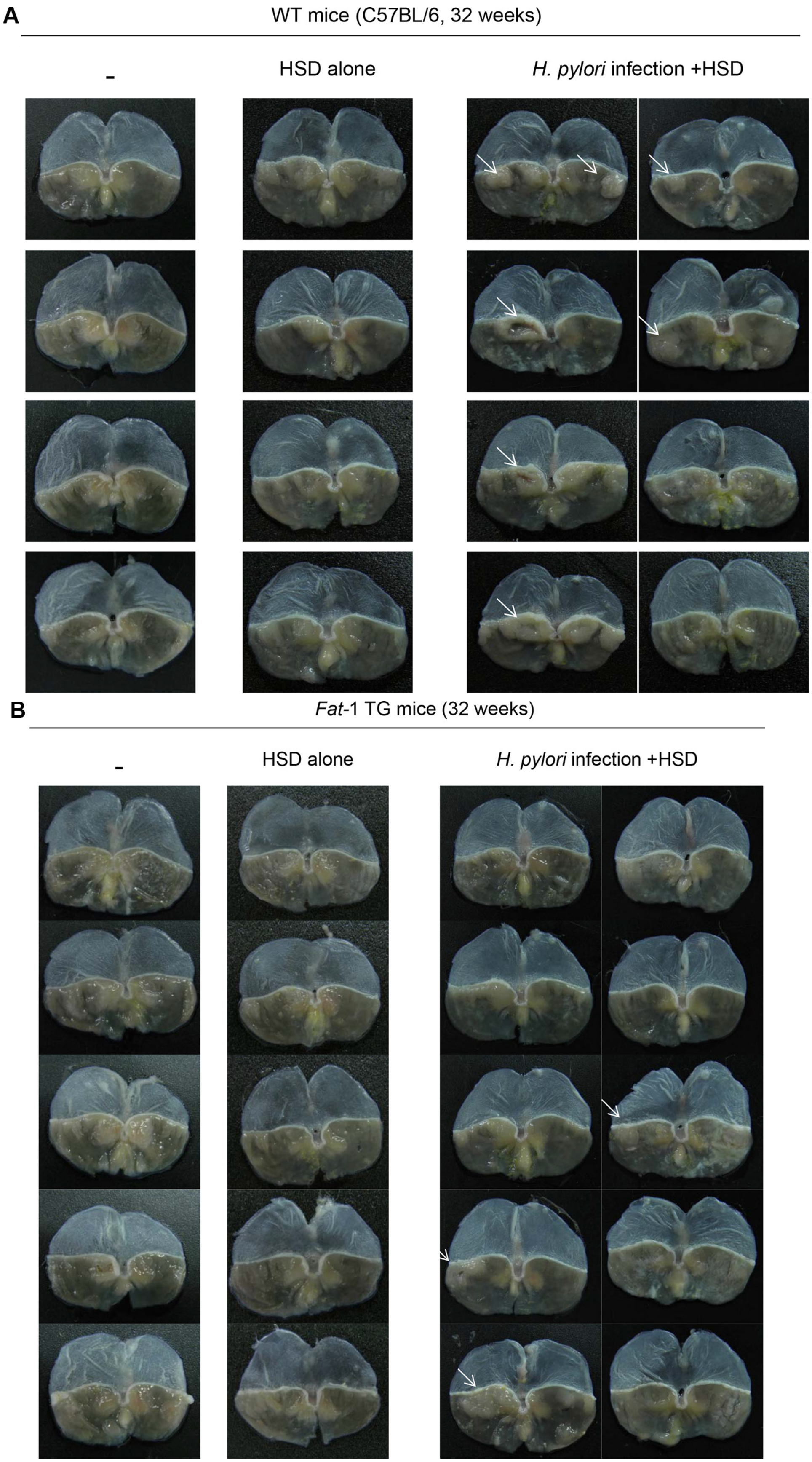

Supplementary Figure S3: Gross photographs of whole mice stomach according to group observed at 32 weeks. (**A**) Non-infected vehicle control group, HSD alone, and H. *pylori* infection of 32 weeks WT mice. (**B**) Non-infected vehicle control group, HSD alone, and H. *pylori* infection of 32 weeks *Fat-1* TG mice.

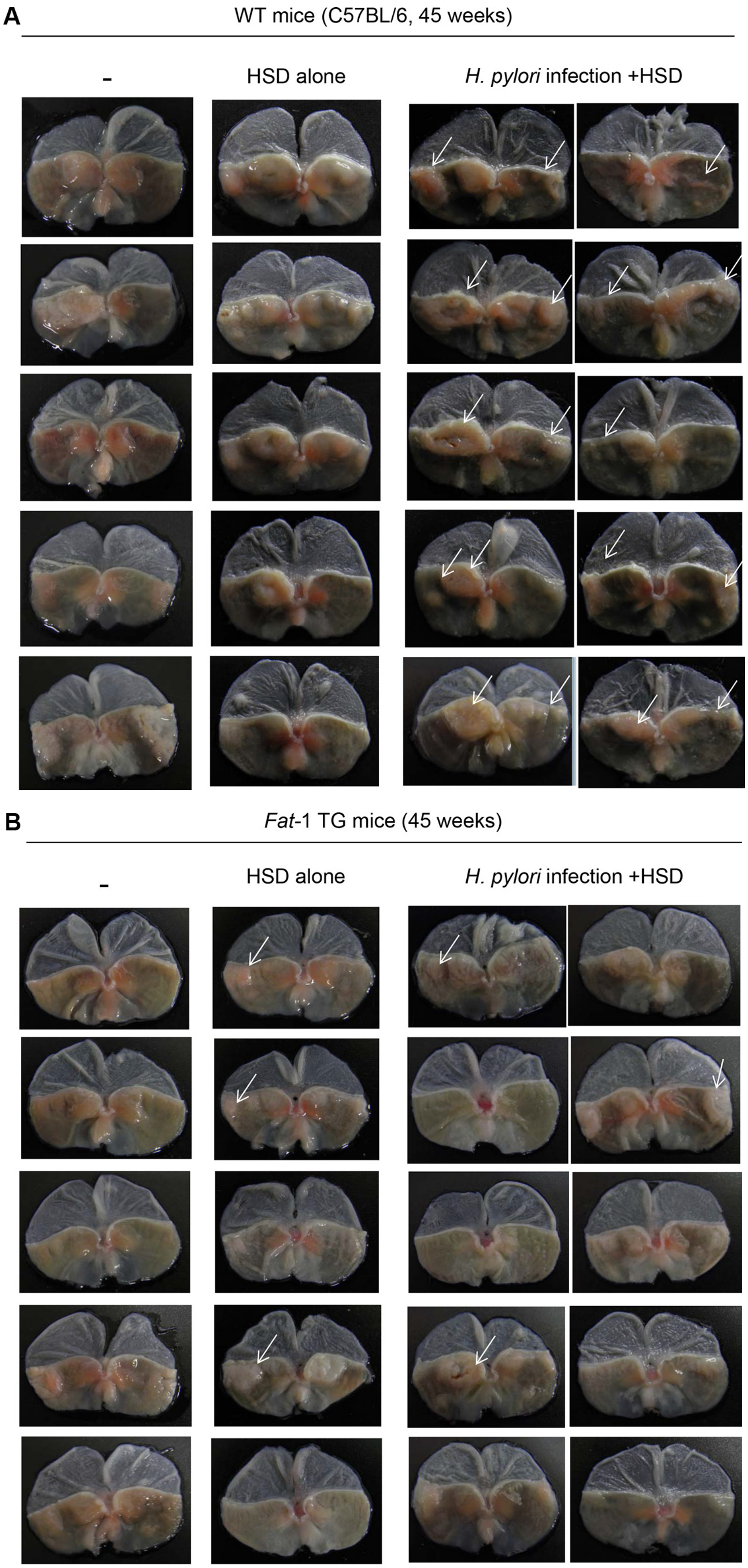

Supplementary Figure S4: Gross photographs of whole mice stomach according to group observed at 45 weeks. (**A**) Non-infected vehicle control group, HSD alone, and H. *pylori* infection of 45 weeks WT mice. (**B**) Non-infected vehicle control group, HSD alone, and H. *pylori* infection of 45 weeks *Fat-1* TG mice.
